# Resistance to Fracture of Dental Roots Obturated with Different Materials

**DOI:** 10.1155/2015/591031

**Published:** 2015-02-08

**Authors:** Berkan Celikten, Ceren Feriha Uzuntas, Kamran Gulsahi

**Affiliations:** ^1^Department of Endodontics, Faculty of Dentistry, Ankara University, Ankara, Turkey; ^2^Department of Endodontics, Faculty of Dentistry, Başkent University, Ankara, Turkey

## Abstract

The aim of this study was to compare the vertical fracture resistance of roots obturated with different root canal filling materials and sealers. Crowns of 55 extracted mandibular premolar teeth were removed to provide root lengths of 13 mm. Five roots were saved as negative control group (canals unprepared and unfilled). Fifty root canals were instrumented and then five roots were saved as positive control group (canals prepared but unfilled). The remaining 45 roots were randomly divided into three experimental groups (*n* = 15 root/group) and obturated with the following procedures: in group 1, glass ionomer-based sealer and cone (ActiV GP obturation system); in group 2, bioceramic sealer and cone (EndoSequence BC obturation system); and in group 3, roots were filled with bioceramic sealer and cone (Smartpaste bio obturation system). All specimens were tested in a universal testing machine for measuring fracture resistance. For each root, the force at the time of fracture was recorded in Newtons. The statistical analysis was performed by using Kruskal-Wallis and post hoc test. There were no significant differences between the three experimental groups. The fracture values of three experimental and negative control groups were significantly higher than the positive control group. Within the limitations of this study, all materials increased the fracture resistance of instrumented roots.

## 1. Introduction

Vertical root fracture is one of the most serious complications of root canal procedures with an unfavorable prognosis that can occur before, during, or after obturation and often result in tooth extraction [1, 2]. Therefore, one of the objectives of root canal obturation is to reinforce the root canal and increase root fracture resistance [3]. The most commonly used root canal filling material is gutta-percha in combination with sealer [4], but the low elastic modulus of gutta-percha presents little or no capacity to reinforce roots after treatment [5]. The ability of sealer to bond to radicular dentin is advantageous in maintaining the integrity of the sealer-dentin interface during mechanical stresses, thus increasing resistance to fracture [6]. New root canal obturation materials have been developed in an attempt to provide all of the favorable properties [7].

ActiV GP (Brasseler USA, Savanah, GA) comprises glass ionomer-coated gutta-percha (ActiV GP cone) cones that are bondable to intraradicular dentin through the use of glass ionomer sealer (ActiV GP sealer). It is considered to be tertiary monoblock system in which there are 3 interfaces between the bonding substrate and the bulk material core [8].

Recently, a new bioceramic root canal obturation system, EndoSequence BC sealer that is used with EndoSequence BC point (Brasseler USA, Savannah, Georgia; also known as iRoot SP, Innovative Bioceramix, Vancouver, Canada), has been marked. The EndoSequence BC sealer is composed of calcium silicates, calcium hydroxide, calcium phosphate monobasic, and zirconium oxide. The manufacturer indicates that it is injectable, premixed, radiopaque, zero shrinkage, insoluble, hydrophilic (using of moisture in dentinal tubules to initiate and complete its setting reaction), and aluminum-free material [9]. EndoSequence BC points are subjected to a patented process of impregnating and coating each cone with bioceramic nanoparticles. According to the manufacturer's claim; the bioceramic particles found in BC sealer used in conjunction with the bioceramic particles in BC points form a true gap-free seal.

Smartpaste bio (DRFP Ltd., Stamford, UK) is also a new bioceramic root canal sealer. The manufacturer claims that it is biocompatible (will not cause irritation if put through the apex), antibacterial, nonresorbable inside the root canal, requires no mixing and expands slightly upon setting. Smartpaste bio produces calcium hydroxide and hydroxyapatite (the matrix of new bone formation) as byproducts of the setting reaction (http://www.smart-seal.co.uk/). Propoint (DRFP Ltd., Stamford, UK) contain a hydrophilic polymer coating around a central core that expands laterally only upon absorbing water from the tooth. According to the manufacturer's claim; the hydrophilic nature of the cement makes this a perfect companion to use with self-sealing propoint, allowing the point to hydrate and swell to fill any voids.

The objective of the present study was to evaluate and compare the effect of different root canal obturation materials and sealers on the fracture resistance of endodontically treated roots.

## 2. Material and Methods

### 2.1. Tooth Selection, Preparation, and Obturation

A total of 55 extracted human mandibular premolar teeth with similar dimensions at the cementoenamel junction (CEJ) were selected, buccolingual and mesiodistal dimensions of the root canals were measured using a digital caliper. The sample size was determined with power analysis. The teeth were carefully examined under an operating microscope (Zeiss, Oberkochen, Germany) with ×20 magnifications. Teeth with immature apices having root caries or restorations and having root fractures or cracks were excluded from the study. Preoperative radiographs were taken in the mesiodistal and buccolingual directions to confirm the presence of a single canal without previous root canal treatment, resorptions, or calcifications. Crowns of the selected teeth were sectioned at the cementoenamel junction (CEJ) with safe-sided diamond disk, to provide root lengths of 13 mm. Working length was determined 1.0 mm shorter than real root canal length. All the root canals, except those in negative control group (*n* = 5, unprepared and unfilled), were instrumented using crown-down technique by RaCe rotary files up to # 40/0.04 taper (FKG, Dentaire Co., Dental Products, Switzerland). Irrigation was performed with 1 mL 2,5% NaOCl between each instrument. A final rinse with 2 mL 2,5% NaOCl for 1 min, 2 mL 17% EDTA for 1 min, and 10 mL distilled water was performed. Roots dried with paper points and then were randomly assigned into 3 experimental (*n* = 15/each) and positive control (*n* = 5, unfilled) groups.  Table 1 shows composition of sealers used in this study.

In group 1 (ActiV GP sealer and ActiV GP cone), each canal was fitted with a single ISO size # 40, 0.04 taper ActiV GP master cone. ActiV GP glass ionomer sealer was mixed according to the manufacturer instructions. The master ActiV GP cone was then coated with the sealer and gently seated into the canal to its working length and then the cone was removed with a warm excavator, and final vertical compaction was completed with a size 11 plugger to a depth of approximately 1 mm into the canal orifice.

In group 2 (EndoSequence BC sealer and EndoSequence BC Point), each canal was fitted with a single ISO size # 40, 0.04 taper EndoSequence BC point. BC sealer was injected through the intracanal tip to fill the apical part of the canal, and the tip was then slowly with drawn while injecting the sealer until complete filling of the canal. The BC point was then introduced into the canal up to working length. Removal of excess cone and final vertical compaction were accomplished in the same manner as group 1.

In group 3 (Smartpaste bio sealer and Propoint), each canal was fitted with a single ISO size # 40, 0.04 taper propoint cone. Smartpaste bio sealer was injected through the intracanal tip to fill the coronal part of the canal. The propoint was then coated with the sealer and gently placed into the canal to its working length. Removal of excess cone and final vertical compaction were accomplished in the same manner as group 1.

The quality of the fillings as confirmed with radiographs. Canals that had not been adequately filled or specimens with cracks were dismissed and replaced by a new sample.

The coronal access of specimens was filled with a temporary filling material (Cavit; 3M ESPE, Seefeld, Germany). All teeth were stored at 37°C and 100% humidity for 2 weeks to allow the sealers to set completely.

### 2.2. Preparation for Fracture Resistance Test

Four millimetres of the apical root ends were vertically embedded into an acrylic tube (13 mm height and 15 mm diameter) with an autopolymerisable acrylic resin (Imicryl, Konya, Turkey), leaving 9 mm of each root exposed [10, 11]. The roots were positioned at the centre of the acrylic tube. The temporary filling material was removed and the specimens were mounted on the lower plate of the universal testing machine (Lloyd LRX; Lloyd Instruments Ltd, Fareham, UK). The upper plate of the machine housed a round tip of 6 mm diameter [12], and a compressive loading was applied to the coronal surfaces of roots with a loading rate of 1,0 mm per minute until the fracture occurred. The force required to fracture each root was recorded in Newtons (N). The results were subjected to statistical analysis using Kruskal-Wallis and post hoc Scheffe test [13] to determine the differences between the groups. The level of significance was set at *P* < 0.05.

## 3. Results

**Figure 1:**
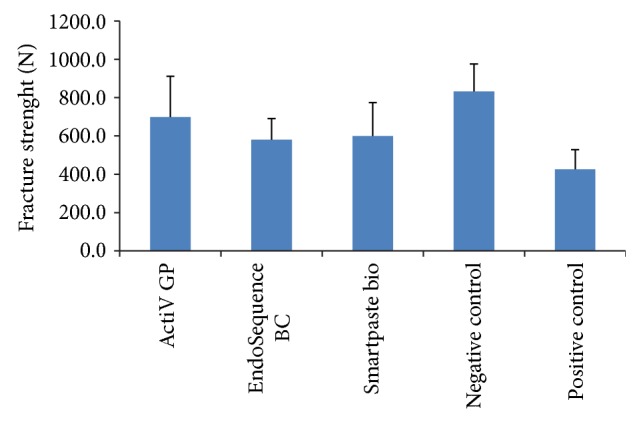
Mean and standard deviation values of fracture strenght for each group (in Newtons).

Table 2 and Figure 1 present the mean values ± standard deviations, 95% Confidence Interval, median, and maximum and minimum of the force required to fracture the roots. While the negative control (unprepared/unfilled) group revealed hight fracture strength (831.9 N), the weakest force required to fracture the roots was seen in the positive control (prepared but unfilled) group (425.9 N). The mean values of experimental groups were 698.6 N, 580.8 N, and 599.1 N for groups 1, 2, and 3, respectively. There was no statistically significant difference in fracture resistance between all experimental groups (*P* > 0.05). Root filled with the ActiV GP sealer + ActiV GP cone showed higher, but not significantly different, fracture values than those filled with EndoSequence BC sealer + EndoSequence BC point and Smartpaste bio sealer + propoint cone (*P* > 0.05). The fracture values of the experimental teeth and negative control group were significantly higher than the positive control group (*P* < 0.05). On the other hand, while there were no significant differences in fracture resistance between group 1 and negative control group (*P* > 0.05), group 2 and group 3 showed the lower mean values for fracture than the negative control group (*P* < 0.05).

## 4. Discussion

One of the most important stages of root canal procedures is adequate obturation of the root canal system after biomechanical preparation. Filling material has a potential to strengthen the root structure and increases fracture resistance of tooth [14]. In order to standardize, roots with similar size, length, and dimensions were used in the study [12, 15]. To standardize the apical diameter of the root canals, size 40/0.04 taper RaCe rotary master file was used in all groups. Preparation of root canal with rotary systems results in a more rounded cross section that may have a positive effect on stresses and force distribution within the root canal during filling [16]. The use of EDTA have some weakening effect on the dentin but this impact can be reduce by using low concentration and exposure time of EDTA [17]. Moreover, low surface tension of EDTA allows it to easily flow into the dentinal tubules and removes the smear layer up to a depth of 2.5–4 *μ*m [18, 19]. After the removal of smear layer, there was an alteration in the surface energy allowing the root canal sealer to flow and adapt more easily, enhancing its adhesion to the root canal wall, thereby increasing sealing efficiency [19, 20]. To neutralize the effects of irrigating solutions, distilled water was used as a final rinse.

Fracture resistance of different obturating systems was evaluated. In the present study, a single-cone obturation technique was used because it excluded both the excessive dentin removal required to facilitate the plugger's insertion during vertical compaction and the wedging forces of the spreaders during lateral compaction [21]. According to the results of this in vitro study (Table 2), the fracture values of the positive control group were significantly lower than the experimental and negative control groups (*P* < 0.05). This result also agrees with a many previous study and can be explained preparation of root canals weakened the roots as the amount of remaining dentin thickness was reduced, and there was no filling material to reinforce tooth structure [9, 12]. In the current study, EndoSequence BC sealer + BC point and Smartpaste bio sealer + propoint cone showed significantly different low mean values for fracture than the negative control group (*P* < 0.05). According to the information provided by the manufacturer, root canal sealers based on bioceramic or calcium silicate needs water for setting and uses the moisture within the dentinal tubules to initiate and complete its setting reaction. The moisture in the dentinal tubules might not be enough for setting these materials, which might account for the lower resistance to fracture of the roots obturated with bioceramic sealers (EndoSequence BC and Smartpaste bio) than unprepared and unfilled teeth. In contrast, a few previous studies showed that the fracture resistance of root treated teeth with bioceramic sealer (iRoot SP and EndoSequence BC sealer) was not significantly difference with the control group of intact roots [9, 12]. These differences could be attributed to the study design (e.g., combined use of iRoot SP and ActiV GP, using of Protaper Ni-Ti rotary file and F3 master gutta-percha cone). In the present study, glass ionomer-based obturation system indicated higher, but not significantly different, fracture values than those filled with two bioceramic root canal filling systems. On the other hand, no differences in fracture strength were observed between roots filled with ActiV GP system and negative control group. This could be related to the tertiary monoblock system in which there are 3 interfaces between the bonding substrate and the bulk material core. According to the manufacturer, placing glass ionomer particles into the gutta-percha and then coating the cone with glass ionomer to a thickness of two microns allows the glass ionomer sealer to directly bond to it. Thus, combined using of ActiV GP and its glass-ionomer sealer produce a true monoblock. Several studies have reported the superior bonding of ActiV GP to root canal dentin [22, 23]. In contrast, Ghoneim et al. [21] showed that the fracture resistance of root filled with ActiV GP sealer and ActiV GP cone was lower than the negative control group. This difference could be attributed to the study design. Clinical long-term studies are necessary to support the confident use of these materials.

## 5. Conclusions

Under the condition of this in vitro study, filling with glass ionomer-based obturation system showed resistance to fracture similar to sound tooth; however, all the obturation materials used in the present study increased the fracture resistance of instrumented root canals.

## Figures and Tables

**Table 1:** Compositions of obturation materials used in this study.

Obturation material	Composition
ActiV GP sealer	Powder: barium alimunasilicate glass powder, dried polyacrylic acid Liquid: polyacrylic acid, tartaric acid

ActiV GP cone	Glass ionomer-coated gutta-percha (2 *μ*m thickness)

Endosequence BC Sealer	Zirconium oxide, calcium silicates, calcium phosphate monobasic, calcium hydroxide, filler and thickening agents

Endosequence BC point	Coating each cone with bioceramic nanoparticles.

Smartpaste bio	Zirconium oxide, tricalcium silicate, dicalcium silicate, calcium hydroxide, filler and thickening agents

Smartpaste bio propoint	Radiopaque core coated with a hydrophilic polymer

**Table 2:** Mean ± standard deviation, 95% Confidence Interval, median, and maximum and minimum values of fracture strength for each group (in Newtons).

Groups	*N*	Mean ± SD	95% Confidence Interval		Median	Max	Min
			Lower bound	Upper bound			
ActiV GP sealer + GP cone	15	698.6 ± 212.8^a,b^	580.7	816.5	708.7	1087.2	377.4
EndoSequence BC sealer + BC point	15	580.8 ± 109.6^a^	520.1	641.5	571.4	883.4	454.1
Smartpaste bio sealer + Propoint	15	599.1 ± 174.6^a^	502.4	695.8	588.0	947.2	368.5
negative control	5	831.9 ± 143.7^b^	653.5	1010.4	784.0	1073.2	692.8
positive control	5	425.9 ± 101.9^c^	299.2	552.5	439.2	543.5	284.0

SD: standard deviation.

Values with the same superscript are not statistically different (*P* > 0.05).
